# The Alkaloidal Treatment of Pneumonia

**Published:** 1912-01

**Authors:** 


### The Alkaloidal Treatment of Pneumonia.

Dr. William J. Johnson of Uxbridge, Mass., says in the *Journal of Therapeutics and Dietetics* that alkaloidal medication in pneumonia is a protest to the orthodox views both on the course of the disease and concerning the value of drugs. The adherents of positive therapeutics are convinced that the course of the disease can be so largely modified, by appropriate medication, that all and, indeed, many of the expected symptoms and conditions do not appear. Alkaloidal medication strikes at the root of the trouble, endeavors to foresee and prevent the changes that naturally appear in a typical case of pneumonia and does not wait for the symptoms to develop and the results to be manifest. For fever we use aconitine, 1-134 grain, one granule every fifteen minutes, half hourly or hourly, until lowering of pulse rate, reduction of temperature, sweating and lessened pain occurs. Combined with this we use strychnine, 1-134 grain, and digitalin, 1-67 grain, for its tonic action and supporting the heart. The other important remedies are emetine, 1-67 grain, and codeine, 1-67 to 1-12 grain hourly to relax spasm, control nervous symptoms, quiet and loosen the cough. As the fever subsides and the cough becomes looser, calcium sulphide, $\frac{1}{6}$ grain every two hours, is such a valuable and important remedy that it should always be used, and its action will not disappoint the careful user. After the acute symptoms have subsided, glonoin, 1-250 grain, is a most valuable remedy, sustaining the vitality wonderfully, prompt and reliable in its action. These are our standard remedies for this disease. They offer a definite amount of a single distinct alkaloid to counteract a definite disease with structural lesions. Their action must be carefully watched, must be changed or modified by the course of the disease, and they produce results at once marked, rapid and permanent.

It goes without reason that the nutrition and hygiene of the patient requires attention, nor are external applications to be despised. The alkaloidal remedies are so positive in their action that we can dispense with alcohol, which at best is an uncertain and treacherous ally.

